# PIEZO1 mechanoreceptor activation reduces adipogenesis in perivascular adipose tissue preadipocytes

**DOI:** 10.3389/fendo.2022.995499

**Published:** 2022-08-31

**Authors:** C. Javier Rendon, Emma Flood, Janice M. Thompson, Miguel Chirivi, Stephanie W. Watts, G. Andres Contreras

**Affiliations:** ^1^ Department of Large Animal Clinical Sciences, Michigan State University, East Lansing, MI, United States; ^2^ Department of Pharmacology and Toxicology, Michigan State University, East Lansing, MI, United States

**Keywords:** perivascular adipose tissue (PVAT), hypertension, PIEZO1, preadipocytes, adipogenesis

## Abstract

During hypertension, vascular remodeling allows the blood vessel to withstand mechanical forces induced by high blood pressure (BP). This process is well characterized in the media and intima layers of the vessel but not in the perivascular adipose tissue (PVAT). In PVAT, there is evidence for fibrosis development during hypertension; however, PVAT remodeling is poorly understood. In non-PVAT depots, mechanical forces can affect adipogenesis and lipogenic stages in preadipocytes. In tissues exposed to high magnitudes of pressure like bone, the activation of the mechanosensor PIEZO1 induces differentiation of progenitor cells towards osteogenic lineages. PVAT’s anatomical location continuously exposes it to forces generated by blood flow that could affect adipogenesis in normotensive and hypertensive states. In this study, we hypothesize that activation of PIEZO1 reduces adipogenesis in PVAT preadipocytes. The hypothesis was tested using pharmacological and mechanical activation of PIEZO1. Thoracic aorta PVAT (APVAT) was collected from 10-wk old male SD rats (n=15) to harvest preadipocytes that were differentiated to adipocytes in the presence of the PIEZO1 agonist Yoda1 (10 µM). Mechanical stretch was applied with the FlexCell System at 12% elongation, half-sine at 1 Hz simultaneously during the 4 d of adipogenesis (MS+, mechanical force applied; MS-, no mechanical force used). Yoda1 reduced adipogenesis by 33% compared with CON and, as expected, increased cytoplasmic Ca2+ flux. MS+ reduced adipogenesis efficiency compared with MS-. When *Piezo1* expression was blocked with siRNA [si*Piezo1*; NC=non-coding siRNA], the anti-adipogenic effect of Yoda1 was reversed in si*Piezo1* cells but not in NC; in contrast, si*Piezo1* did not alter the inhibitory effect of MS+ on adipogenesis. These data demonstrate that PIEZO1 activation in PVAT reduces adipogenesis and lipogenesis and provides initial evidence for an adaptive response to excessive mechanical forces in PVAT during hypertension.

## Introduction

The perivascular adipose tissue (PVAT) is an integral part of blood vessels. As such, changes in its structure and function are part of the pathogenesis of hypertension and other cardiovascular diseases (1, 2). Like other adipose tissues (AT) depots, PVAT is composed of adipocytes and a stromal vascular fraction containing immune, vascular, endothelial, neural, and stem cells of different lineages (3). PVAT is present in most blood vessels except the brain (4–6). The importance of the PVAT on vascular function is related in part to its capacity to modulate the contractile function of the vasculature. In addition, many of the vasoactive factors secreted by PVAT are adipokines (e.g., adiponectin). Thus, maintaining a healthy population of adipocytes is essential to support adequate vascular function.

PVATs are unique among AT depots as they are exposed to continuous and synchronous mechanical forces exerted by blood flow, including tensile stress and cyclic strain (reviewed by Hayashi and Naiki (7)). During hypertension, as blood pressure rises, these mechanical forces induce changes in the physiology of cellular components of the vascular tunicas intima, media, and adventitia. This process is known as vascular remodeling and includes alterations such as cellular hypertrophy and hyperplasia and collagen deposition in different tunicas (8–10). The direct effects of this process on the vasculature include reduced vessels’ elasticity and compliance and increased stiffness (11, 12). For example, the thoracic aorta in hypertensive patients has lower elasticity, increased vascular thickness, and fibrosis of its anatomical layers compared to normotensive patients (13, 14). The effects of mechanical forces on the adipogenic and lipogenic capacity of PVAT preadipocytes are currently unknown. However, there are reports on cellular responses to mechanical stimuli in cell lines and stromal vascular fraction (SVF)-derived preadipocytes harvested from non-PVAT depots. In the 3T3-L1 fibroblast, equibiaxial stretching above 9% promotes lipogenesis, but its effects on adipogenesis are not described. In the human adipogenic cell line SGBS, compressive force inhibits adipogenesis by suppressing the expression of its master regulator PPARγ. Reports from primary cell lines are scarce and do not include pharmacological and mechanical stimulation of mechanoreceptors (15–17).

Recent studies demonstrate that adipocytes express mechanoreceptors and, therefore, could sense mechanical forces through these specialized proteins (18). Among these, PIEZO1 is present in murine subcutaneous and visceral fats (18, 19) and aortic and mesenteric PVAT (20). This mechanosensor is a transmembrane protein capable of transforming a physical stimulus into a chemical signal. PIEZO1 is a non-selective Na+, K+, and Ca+ permeable channel (21–26). This mechanoreceptor is essential for regulating sensation, touch, and blood pressure (27). PIEZO1 appears to play a role in the differentiation process of resident progenitor cells such as neural stem cells and human mesenchymal stem cells (28, 29). In visceral AT, Offermanns and colleagues showed that during high-fat diet-induced adiposity, the enlargement of lipid droplets in non-PVAT adipocytes generates a stretch force that activates PIEZO1, indicating the capacity of these cells to sense mechanical forces (18). However, it is unknown if PVAT preadipocytes express *Piezo1*, how PIEZO1 activation alters PVAT adipogenic and lipogenic responses, and if PIEZO1 activity could be a mechanism triggering PVAT remodeling during cardiovascular diseases.

This study evaluated the role of the mechanosensor PIEZO1 on PVAT adipogenic processes. We hypothesized that activation of PIEZO1 in PVAT preadipocytes limits their adipogenic capacity. We identified that PVAT preadipocytes express the gene encoding and the protein PIEZO1. Pharmacological and mechanical activation of PIEZO1 reduced adipogenesis efficiency. Our data demonstrate that in PVAT, PIEZO1 activation regulates adipogenesis, possibly linking hypertension with the loss of adipocyte PVAT populations.

## Materials and methods

### Animals

Male Sprague-Dawley rats of 8-10 weeks (Charles River Laboratories, Inc., Portage, MI, RRID: SCR_003792) were housed in a temperature-controlled room at 22°C, with 12:12-h light-dark cycles and environmental enrichment using standard cages. Animals were fed a regular chow diet with distilled water ad libitum. All animal procedures were approved by the MSU Institutional Animal Care and Use Committee and followed the “Guide for the Care and Use of Laboratory Animals,” 8th edition (30). Rats were anesthetized with an intraperitoneal injection of 60-80 mg/kg of pentobarbital. Deep anesthesia was verified by lack of paw pinch and eye-blink reflexes, and death was assured by pneumothorax.

### Preadipocyte isolation and culture

The thoracic aorta, including its perivascular adipose tissue (APVAT), was dissected and then immersed in Krebs-Ringer Bicarbonate Buffer (KRBB) containing NaCl 135 mM; KCl 5 mM; MgSO4 1 mM; KH2PO4 0.4 mM; Glucose 5.5 mM; HEPES 20 mM (pH 7.4) (Teknova, Cat N° H1030) and supplemented with 100 units/mL of penicillin; 100 µg/mL of streptomycin, 0.25 µg/mL of Amphotericin B and 50µg/mL of Gentamicin. PVAT preadipocytes were isolated as previously described (5, 31). The APVAT was separated from the thoracic aorta under a dissection stereoscope, and ~50mg fragments were minced in 1-3 mm pieces and then digested for 1 h at 37°C in a rotisserie incubator using 0.5mg/mL of Liberase™ TL (Roche diagnostics, Cat N° 5401020001) dissolved in Hanks’ balanced salt solution supplemented with 4% BSA (Fisher, Cat N° BP9706-100) and 10 mM HEPES. Digested material was filtered through 70 µm cell strainers (Corning, Cat N° 22363548) and then centrifuged at 37°C for 5 min at 300 x g to remove the buoyant cells (adipocytes) from the stromal vascular fraction (SVF) containing preadipocytes. Pellets were resuspended in RBC lysis buffer 1X (Biolegend, Cat N° 420301), incubated at room temperature for 5 min, and then centrifuged at 37°C for 5 min at 300 x g; pellets were resuspended in MesenPRO RS™ (ThermoFisher, Cat N° 12746012) with 2% Fetal Bovine Serum (FBS) (Corning, Cat N° 35-016-CV) and plated in T25 flasks (Sigma, Cat N° SIAL0639) and cultured at 37°C with 5% CO2. Cells were expanded as previously described using preadipocyte medium (PAM) containing 10% FBS, Dulbecco’s Modified Eagle’s Medium/F12, 44.05 mM sodium bicarbonate (Corning, Cat N° 61-065-RO), 100 µmM ascorbic acid, (Sigma-Aldrich, Cat N° A4544-100G), 33 µM biotin (Sigma-Aldrich, B4501-1G), 17 µM pantothenate (Sigma-Aldrich, Cat N° P5155-100G), 1% L-glutamine (Gibco, Cat N° 25030-081), 1 µg/mL amphotericin (Sigma-Aldrich, Cat N° A-2942) 10 µg/mL ampicillin (Sigma-Aldrich, Cat N° A0166-5G) and 20mM HEPES with replacement every 2 days (32).

### Immunohistochemistry

To assess the expression of PIEZO1, preadipocytes and tissue sections were processed for immunohistochemistry as described with some modifications (5). First, preadipocytes cells were seeded on Ibidi µ-slide 8 well (Ibidi GmbH, Cat N° 80822) and incubated overnight to allow cell adherence; after washing with PBS were fixed with 4% of paraformaldehyde in PBS. On the other hand, tissue sections from the thoracic aorta that included PVAT were harvested, kidney samples were used as a positive control for PIEZO1 expression. Tissues were fixed in 4% formalin, embedded in paraffin, and then sectioned into 4–5 μm by the Michigan State University Investigative Histopathology Laboratory. Slides with tissues and cells were incubated in a species-specific serum (1.5% goat serum in PBS-TX; Vector Laboratories, Burlingame, Canada) and then incubated overnight with PIEZO1 primary antibody (1:600, Alomone, Cat N° APC-087, RRID : AB_2756743). The next day, samples were washed with PBS and incubated with AlexaFluor 488 goat anti Rabbit (ThermoFisher, Cat N° A11008, RRID: AB_143165) for 30 mins at room temperature. A second wash was performed, and cells were counterstained with VectaShield^®^ HardSet™ with DAPI (Vector Laboratories, Cat N° H-1500-10). Images were captured with a Nikon Digital Sight DS-Qil camera in a Nikon Eclipse Ti inverted microscope at an x20 magnification with a Lumencore LED light source and Nikon NIS Elements BR 3.00 software. Each photograph is a combination of DAPI (Nuclei marker) and FITC (PIEZO1) channels and standardized for true fluorescence based on the control FITC for that specific tissue section that was then embedded into the image and carried through all image analysis.

### Adipogenesis experiments

Preadipocytes were seeded in six well-plates and induced to differentiate into adipocytes in standard adipogenic media alone, as previously described by our group (31), in the presence of the PIEZO1 agonist Yoda1 10 µM (Tocris, Cat N° 5586), a concentration reported in different cell types (18, 29, 33–36). Adipogenesis was evaluated using Bodipy 493/503 (ThermoFisher Cat N° D3922), a neutral lipid staining, and the nuclear stain NucSpot^®^ Live 650 (Biotium, Cat N° 40082) and reported as Bodipy fluorescence intensity/nuclei count using long-term live-cell imaging IncuCyte^®^ S3 system. Cultured cells were imaged every 6 hours during 4 days in culture. Quantification of cell images after 4 days was performed using IncuCyte ZOOM™ software.

### Viability and apoptosis assay

Viability and apoptosis assays were performed according to the manufacturer’s specifications. Briefly, viability was determined in preadipocytes exposed to 10 µM Yoda1 (Tocris, Cat N° 5586) in adipogenic media for 4 days. Negative control of dead cells was made with 0.1% of saponin (Alfa Aesar, Cat N° J63209) in 1X PBS for 10 mins before collection time, all conditions were incubated at room temperature with Calcein AM (Biotium, Cat N° 30002) during 45 mins. Fluorescence was measured in a microplate reader (BioTek, Synergy H1M). Apoptosis was calculated detecting Caspase 3/7 activity (Biotium, Cat N° 10403), preadipocytes were exposed to different concentrations of Yoda1 during 4 days of adipogenic induction, and with 0.2 µg/mL of Doxorubicin HCl (TCI, Cat N° D4193) used as a positive control of apoptosis, fluorescence is reported as relative fluorescence units (RFU).

### RNA isolation and purification

RNA was extracted using the Maxwell^®^ RSC simplyRNA cells kit (AS1390, Promega, Madison, WI) as reported previously (32). Cultured cells were homogenized in 1-Thioglycerol/Homogenization Solution (Maxwell^®^ Cat N° Z305H) and vortexed for 15 sec. The cell lysate was mixed with 200 μL lysis buffer (Maxwell^®^ Cat N° MC501C) and vortexed for 15 sec. The 400 μL cell lysates were transferred into the sample well of the Maxwell^®^ RSC Cartridge. 5 μL of DNase I solution were added in well #4 of the Maxwell^®^ RSC Cartridge to eliminate genomic DNA. The RNA was automated extracted in Maxwell^®^ RSC Instrument (Promega, Madison, WI, USA) following the manufacturer protocol. The purity, concentration, and integrity of mRNA were evaluated using a NanoDrop One^©^ spectrophotometer (Thermo Scientific, Cat N° 840274200). All samples had a 260:280 nm ratio between 1.9 and 2.1 and the RNA integrity number > 7.

### cDNA synthesis and qualitative PCR

Reverse transcription was performed with 500 ng of RNA in 20 μL of reaction volume containing 4 μL of qScript cDNA SuperMix (95048-500, Quantabio, Beverly Hills, CA, USA). The reverse transcription conditions were 5 minutes at 25°C, 30 minutes at 42°C, and 5 minutes at 85°C; cDNA was stored at -20°C.

**Figure 1:**
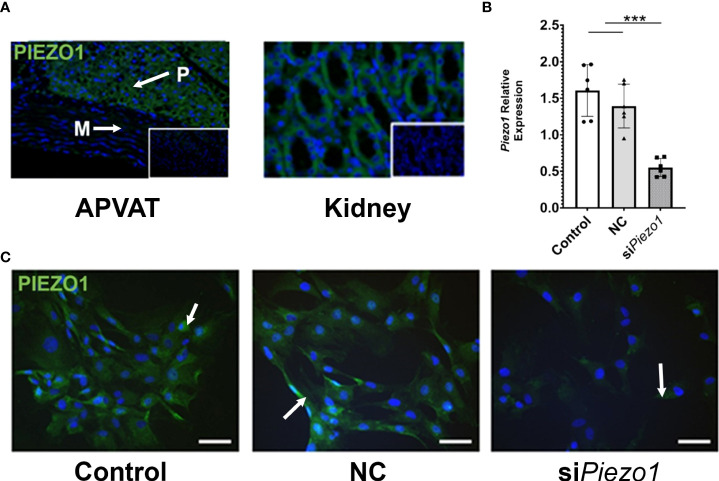
PVAT and preadipocytes express PIEZO1. **(A)** PIEZO1 staining of thoracic aorta PVAT and kidney. White arrows indicate positive cells. DAPI is the nuclear stain. Inserts are sections without primary antibody. P=PVAT, M=Media vascular layer. Images are representative of n=4. **(B)** Piezo1 expression in APVAT preadipocytes treated with *Piezo1* siRNA (si*Piezo1*), non-coding si*Piezo1* (NC), and non-treated (Control). **(C)** PIEZO1 staining of cells in B are representative of n=6. Scale bars = 50 µm. Bars are means ± SEM, Bars with *** (P<0.001) are different relative to the expression of *Actb*.

Transcriptional studies were performed using the the Quan Studio 7 Flex System (Applied Biosystems, MA, USA) and the high-throughput qPCR instrument Wafergen Smartchip (Takara Bio, Mountain View, CA, USA) conducted with Taqman or SYBR gene expression primers commercially available or designed from murine sequences and synthesized (IDT Technologies (Coralville, IA). For PCR data in **Figure 1** samples were assayed in duplicate. Each 20 µL of PCR reaction contained 1X of PerfeCTa Fast Mix II (Quantabio, Cat N° 95119-012), Taqman assays (**Suppl Table 1**) were used at 1X, and 4 ng/µL of sample cDNA. For all other experiments, the samples were assayed in duplicate. Each 20 µL of PCR reaction contained 1X of PerfeCTa Fast Mix II (Quantabio, Cat N° 95119-012), Taqman assays (**Suppl Table 1**) were used at 1X, and 4 ng/µL of sample cDNA. For all other experiments, the samples were assayed in duplicate; each 100 nL PCR reaction contained 1X of LightCycler 480 SYBR Green Master Mix (Roche), 200 nM of primer assays, and 1.5 ng/μL of sample cDNA. A non-reverse-transcriptase control and no-template control examined the DNA contamination and primer-dimer formation in the assay reaction. Primer sequences are reported in **Suppl Table 1**. The cycling conditions for Taqman assays included: initial enzyme activation at 95°C for 20 sec, 40 cycles of denaturation at 95 °C for 1 sec, and annealing/extension at 60 °C for 20 sec. For SYBR green assays, the cycler conditions included: initial enzyme activation at 95°C for 10 min, 45 cycles of denaturation at 95°C for 10 sec, and annealing/extension at 60°C for 60 sec, followed by a melting curve analysis of 65-95°C with 0.5°C increments 5 sec per step. The housekeeping genes with the lowest pairwise variation value were selected, including, Eif3k (eukaryotic translation initiation factor 3 subunit k), Rps29 (ribosomal protein s29), and B2m (Beta-2-microglobulin). The expression of target genes was normalized against the geometric mean of selected housekeeping genes as described by (37).

### Short interference RNA

To assess the effects of PIEZO1 activation on adipogenesis, we inhibited the expression of the protein by using short interference RNA (siRNA) following a human preadipocyte protocol with some modifications (38). In brief, 21.000/cm2 preadipocytes were plated at 70% confluency. Cells were incubated overnight in αMEM (Corning, Cat N° 50-010-PB), 5% FBS, Hiperfect transfection reagent (Qiagen, Cat N° 301704), and 40 nM of three combined siRNA sequences targeting *Piezo1*, a non-coding siRNA (NC), or a siRNA targeting *Hprt* (**Suppl Table 1**). All sequences were designed by IDT Technologies (Coralville, IA). Control without the addition of transfection reagent was also included. After incubation, the medium was replaced with preadipocyte media, and cells were allowed to proliferate for 72 h. The fluorescence dye TYE 563 was used to optimize the concentration of sequence and transfection reagent.

### Calcium influx experiments

Cellular calcium trafficking was determined using the indicator Fluo-4 AM Ester (Biotium Cat ° 50018). Cells were washed with Krebs-Ringer-HEPES-Glucose Buffer (KRH-glc) that includes 25mM of glucose and then loaded with 5 µM of Fluo-4 AM in KRH-glc + 0.5% bovine serum albumin (BSA) for 30 mins at room temperature protected from light. Two washes removed extracellular fluorophore with KRH-glc + 0.5%BSA, then measured all the conditions using the same buffer. To evaluate the kinetics of calcium, Yoda1 (10µM; 5586, Tocris, Bristol, UK) was added after the basal reading (0 min), DMSO, and 5µM of Ionomycin (Biotium Cat N° 59007) were used as negative and positive control respectively. The fluorescence intensity excited at 488 nm and emitted at 526 nm is proportional to the cytosolic free calcium concentrations, using microplate reader samples were measured in triplicates (BioTek, Synergy Cat N° H1M).

### Mechanical stretch experiments

Preadipocytes cells were seeded in collagen type 1-coated Bioflex 6-well plates (Flexcell International, Cat N° BF-3001C) at 40,000 cells/cm^2^. Cells were transfected with siRNA and NC sequences as described above; after they reached full confluency 3 d post-transfection were induced to differentiate into adipocytes as previously described. Mechanical stretching was applied simultaneously using the FX-6000 Tension System. This computer-driven instrument creates strain conditions with vacuum pressure to deform the cells on the flexible, matrix-bonded surface of the Bioflex plates. The mechanical stress (MS+) was set up at a rate of 1 Hz with 12% elongation in a half-sine pattern for 4 d; cells without mechanical stretch (MS-) were used as control. Samples were analyzed for gene expression and adipogenesis efficiency using the Spectrum Image Cytometry System. Briefly, cells were washed with PBS 1X and detached with Trypsin 0.05%, centrifugated two times at 300 x g for 5 mins, and then resuspended in 40µl of dye master mix (2X ViaStain Far Red (Nexcelom Cat N° CS1-V0010-1) + 10µM of Bodipy 493/503 in PBS 1X). Images in triplicate were obtained in Spectrum 5 software and analyzed using ImageJ software, and adipogenesis efficiency was determined as Bodipy fluorescence count/nuclei count.

### Statistical analysis

Data were analyzed by one- or two-way ANOVA using JMP (SAS, Cary, NC), SAS 9.4 (SAS Inst, Cary, NC), and GraphPad Software (GraphPad, San Diego, CA). Proc Mixed program was used, and *post hoc* comparisons were performed using Tukey’s adjustments test. Residuals of the model were checked for normal distribution—random effect of the rat within the treatment and mechanical stretch. Statistical significance was set at P ≤ 0.05.

### Declaration and ethical statements

All animal procedures were approved by the MSU Institutional Animal Care and Use Committee and followed the Guide for the Care and Use of Laboratory Animals,” 8th edition (30).

## Results

### Preadipocytes from PVAT express PIEZO1

The PVAT surrounding the thoracic aorta (APVAT) expresses the protein PIEZO1 (**Figure 1A**). To validate the specificity of the PIEZO1 antibody, we used targeted gene knockdown with siRNA in APVAT preadipocytes. *Piezo1* expression was reduced by 70% in cells treated with the siRNA targeting its transcription (si*Piezo1*) compared with those exposed to non-coding siRNA (NC, **Figure 1B**). In addition, preadipocytes treated with si*Piezo1* showed a reduced signal intensity of PIEZO1 compared to untreated cells and NC (**Figure 1C**).

### PIEZO1 activation in PVAT preadipocytes promotes calcium influx

**Figure 2:**
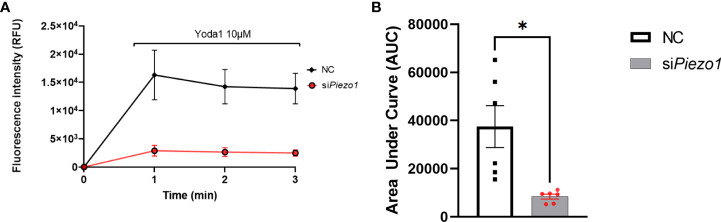
PIEZO1 activation enhances calcium influx in PVAT preadipocytes. **(A)** Fluo-4-loaded preadipocytes treated with non-coding (NC) and si*Piezo1* (siRNA *Piezo1*), the activity of Fluo-4 AM was measured in fluorescence intensity after exposure to 10 µM of Yoda1 starting before min 1. **(B)** Area Under the Curve of fluorescence units of Fluo-4 AM in si*Piezo1* treated cells and NC preadipocytes depicted in **(A)** *P<0.05. representative of n=6.

Treating APVAT preadipocytes with the selective agonist Yoda1 increased Ca2+ flux into the cells treated with NC sequence measured with Fluo-4 AM, a molecule that exhibits fluorescence upon Ca2+ binding. Preadipocytes treated with si*Piezo1* were unresponsive to Yoda1 (**Figure 2**).

### PIEZO1 activation reduces adipogenesis in PVAT preadipocytes

**Figure 3:**
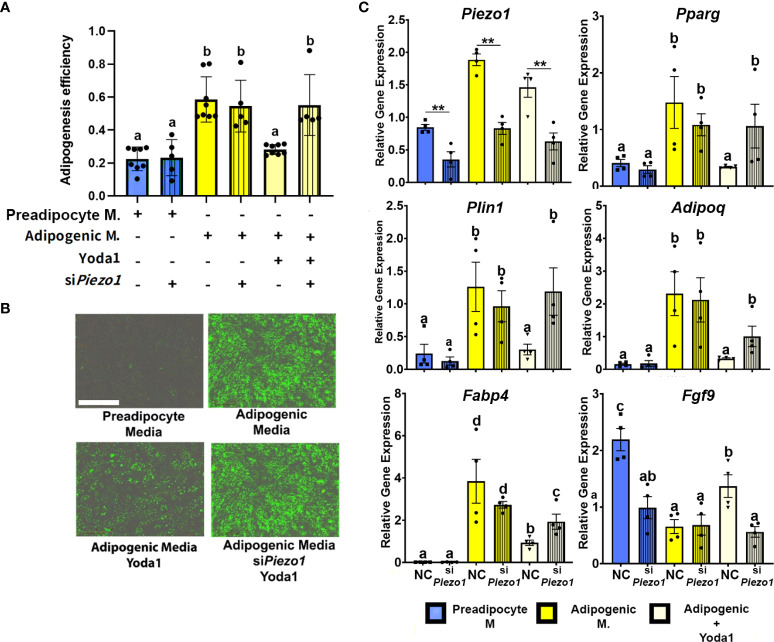
Activation of PIEZO1 reduces adipogenesis in APVAT. Preadipocytes were incubated in preadipocyte or adipogenic media (M.) in the presence of the *Piezo1* activator Yoda1. A subset of cells was treated with siRNA targeting Piezo1 (si*Piezo1*) or non-coding (NC) siRNA. **(A)** Adipogenesis efficiency as calculated by the IncuCyte zoom software (N° of cells with at least one lipid droplet over total # of cells in each well), columns with strips indicate (siPiezo1) n=5-8. **(B)** Representative images of cultured cells. Triglycerides, in green, were stained with Bodipy. Scale bar = 200 microns. **(C)** Expression of Piezo1, adipogenic genes (Pparg, Plin1, Adipoq, and Fabp4) and Fgf9. Values are relative mRNA abundance after normalization with the reference gene Rps29. Bars are means ± SEM; bars with different letters a, b, c, d (P<0.05) or ** (P<0.01) differ. Images in **(B, C)** are representative of n=4.

To evaluate the effect of PIEZO1 activation on APVAT preadipocytes, we treated cells during the first 4 days of adipogenesis induction with the PIEZO1-specific activator Yoda1. Yoda1 at different concentrations during adipogenesis did not affect the viability as determined by Calcein AM a fluorescent molecule emitted by live cells, or induce Caspase 3/7 activity, an enzyme stimulated during apoptosis (**Supplementary Figure 1**). After 4 days of culture in adipogenic media, cells treated with Yoda1 had lower adipogenic efficiency than cells cultured in adipogenic media alone and this was reflected as a reduction in lipid droplet formation (**Figures 3A, B**). Preadipocytes exposed to Yoda1 had reduced expression of critical adipogenic genes such as *Pparg*, *Plin1*, *Adipoq*, and *Fabp4* compared with cells in adipogenic media alone (**Figure 3C**).

A subset of APVAT preadipocytes were treated with si*Piezo1* or NC; this allowed us to reduce the expression of *Piezo1* during 7 days of culture (**Figure 3C**). Silencing the mechanoreceptor abrogated the anti-adipogenic effect of Yoda1, which was reflected in a higher adipogenesis efficiency compared to NC cells treated with Yoda1 (**Figures 3A, B**). si*Piezo1* treated cells cultured in media with Yoda1 had similar expression of adipogenic genes (*Pparg*, *Plin1*, *Adipoq*, and *Fabp4*) to cells treated with NC alone in adipogenic media, while *Fgf9* expression was not increased by Yoda1 when si*Piezo1* was used (**Figure 3C**).

### Cyclic mechanical stretch impairs the adipogenic potential of APVAT preadipocytes regardless of *Piezo1* expression

**Figure 4:**
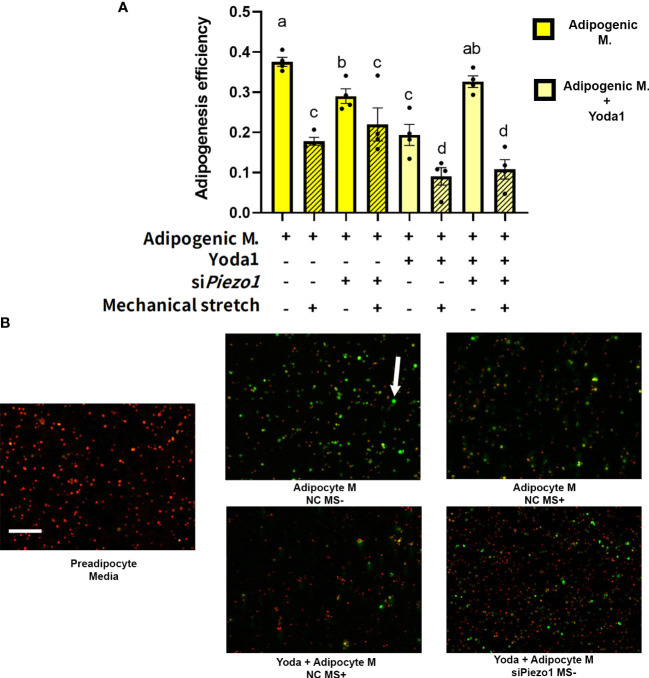
Mechanical stretching affects adipogenesis efficiency. Preadipocytes previously exposed to siPiezo1 (si*Piezo1*+) and NC control (si*Piezo1*-) were induced to become adipocytes in the presence of Yoda 1 (10µM: Yoda1+, 0µM: Yoda1-) and 12% of cyclic elongation (MS+) or 0% (MS-) for 4 days. **(A)** Adipogenesis efficiency calculated by ImageJ (N° cells with at least one lipid droplet over total # of cells per image) analyzed by triplicates. Columns with strips indicate mechanical stretch (MS+). Significant differences are indicated by letters a, b, c, and d (P < 0.05). Bars are means ± SEM. **(B)** Representative images of cultured cells: Triglycerides indicated by the arrow were stained with Bodipy and nuclei with ViaStain Far Red. Images in B are representative of n=4, scale bar = 200 microns.

The next step was to evaluate the effect of direct mechanical stimulation on APVAT preadipocytes adipogenesis. Cells pre-treated with siRNA (si*Piezo1*+) and NC (si*Piezo1*-) sequences were exposed to 4 days of cyclic stretch (MS+) simultaneously during adipogenesis induction (Adipogenic media). Compared to MS-, MS+ reduced adipogenic efficiency in cells cultured in adipogenic media pretreated with NC or si*Piezo1*, and this was reflected as fewer lipid droplets within each cell. A similar response was observed when PIEZO1 was pharmacologically activated with Yoda1 in NC and MS- conditions (**Figures 4A, B**). Yoda1 and MS+ had an additive effect in the reduction of adipogenesis in NC-treated cells. However, si*Piezo1* inhibited the impact of Yoda1 only in MS- cells. si*Piezo1* did not obliterate the effects of mechanical stretching on adipogenesis of cells exposed to Yoda1 and MS+ (**Figures 4A, B**).

Pharmacological and mechanical activation of PIEZO1 altered the expression of some adipogenic and lipogenic gene networks in APVAT. The activation of PIEZO1 by mechanical or pharmacological conditions did not change the expression of mechanoreceptors *Piezo1* or *Piezo2* (**Table 1**). Yoda1 suppressed the lipogenesis-related genes *Dgat1* and *Agpat2* which encode rate-limiting enzymes of triacylglycerol synthesis in adipocytes. Genes associated with extracellular matrix proteins such as *Col6a1* had reduced expression in mechanically stimulated cells, while *Col1a1*, and *Fn1*, were not altered by either chemical (Yoda1) or mechanical activation (MS+) of PIEZO1 (**Table 1**).

**Table 1:** The effect of mechanical and simultaneous pharmacological activation of PIEZO1 on the expression of genes related to mechanoreceptors, adipogenic, lipogenic, fibrogenic, extracellular matrix, and WNT/B catenin pathway.

Gene network	Gene		(MS-)		(MS+)		*P* values		
			Adipogenic M	Yoda1	Adipogenic M	Yoda1	MS	Tx	MS*Tx
Mechanoreceptors	*Piezo1* ^1^	Estimate	1.95	1.73	1.73	1.47	NS^5^	NS	NS
		LCI^2^	1.70	1.48	1.48	1.23			
		UCI^3^	2.20	1.98	1.97	1.72			
	Piezo2	Estimate	2.05	0.34	0.45	1.82	NS	NS	NS
		LCI	0.75	-0.85	-0.74	0.62			
		UCI	3.36	1.53	1.64	3.01			
Lipogenic	Agpat2	Estimate	14.84^a^	6.61^b^	10.97^a^	5.51^b^	NS	<.0001	NS
		LCI	10.54	2.30	6.67	1.20			
		UCI	19.15	10.92	15.28	9.82			
	Dgat1	Estimate	3.66^a^	2.44^b^	2.75^a^	1.62^b^	NS	<.0001	NS
		LCI	2.77	1.55	1.87	0.73			
		UCI	4.54	3.32	3.64	2.50			
Extracellular matrix	Col1a1	Estimate	0.24	0.24	0.25	0.36	NS	NS	NS
		LCI	0.02	0.01	0.03	0.14			
		UCI	0.46	0.46	0.47	0.58			
	Col6a1	Estimate	1.12^a^	1.51^a^	0.88^b^	0.92^b^	<0.05	NS	NS
		LCI	0.76	1.15	0.52	0.56			
		UCI	1.48	1.87	1.23	1.28			
	Fn1	Estimate	0.66	0.66	0.65	0.74	NS	NS	NS
		LCI	0.32	0.32	0.31	0.40			
		UCI	1.00	1.00	0.99	1.08			
Fibroblastic	Fgf10	Estimate	1.45^a^	1.32^a^	1.70^b^	1.81^b^	<0.05	NS	NS
		LCI	0.69	0.55	0.94	1.05			
		UCI	2.21	2.08	2.47	2.57			
	Fgf2	Estimate	0.63	0.77	0.66	0.72	NS	NS	NS
		LCI	0.20	0.34	0.23	0.29			
		UCI	1.06	1.20	1.09	1.16			
	Fgfr1	Estimate	1.43	1.47	1.45	1.48	NS	NS	NS
		LCI	1.06	1.10	1.09	1.11			
		UCI	1.79	1.83	1.82	1.84			
	Fgfr2	Estimate	2.06^a^	1.54^a^	1.37^b^	1.32^b^	<0.01	NS	NS
		LCI	1.67	1.15	0.99	0.94			
		UCI	2.44	1.93	1.76	1.71			
	Fgfr3	Estimate	1.95	2.28	1.63	1.65	NS	NS	NS
		LCI	1.03	1.36	0.71	0.73			
		UCI	2.88	3.21	2.56	2.58			
WNT/β catenin	Wnt16	Estimate	3.88^b^	10.99^a^	5.98^b^	12.61^a^	NS	<0.05	NS
		LCI	-4.70	2.41	-1.86	4.77			
		UCI	12.45	19.57	13.83	20.45			

PVAT preadipocytes (n=6) exposed to 0 (**MS-**) or 12% elongation (**MS+**) in different media conditions (**Tx)**, adipogenic media, and adipogenic media + 10µM Yoda1 (Yoda) for 4 d.

^a-c^ Fold changes without a common superscript within a row represent differences determined by Tukey adjustments of ΔCt values.

Fold change = 2(^-ΔΔCt^); ΔΔCt = ΔCt_calibrator sample_ – ΔCt_target sample_.

^1^Gene expression values were calculated from LSM differences of the ΔCt values (ΔΔCt) normalized to the mean of Eif3k, Rps9, and B2m housekeeping genes.

**^2^LCI**= Lower confidence interval 95%.

**^3^UCI**= Upper confidence interval 95%.

**^4^MS**= Mechanical stretching, **Tx**= treatment.

**^5^NS** = P > 0.10.

As for fibroblast growth factors, MS+ increased *Fgf10*, a gene essential for cell proliferation and tissue repair, while *Fgfr2*, a receptor of the same family, was downregulated. Other genes from this network, *Fgf2*, *Fgfr1*, or *Fgfr3*, were not affected by Yoda1 or MS. In addition, *Wnt16*, a member of the WNT/β catenin pathway, was upregulated by Yoda1 exposure (**Table 1**).

## Discussion

A healthy PVAT is a source of vasoactive molecules, including adiponectin and nitric oxide. During hypertension, PVAT inflammation impairs its capacity to secrete these products (8, 14). A possible mechanism is the reduction of adipocyte populations. Results from the present study suggest that these changes in the cellular populations of PVAT may be related to a decline in the adipogenic potential of preadipocytes driven by the activity of the stretch-activated ion channel PIEZO1. Physical forces generated by blood flow may enhance the calcium influx in preadipocytes through PIEZO1 and ultimately reduce adipogenesis.

### APVAT preadipocytes mechanosense through PIEZO1

Our results indicate that the PVAT of the thoracic aorta is a vascular layer with mechanosensory capacity. First, preadipocytes isolated from APVAT express the mechanotransducer PIEZO1 abundantly, a result that concurs with the evidence found by Miron et al. in PVAT depots (20). Other tissues subjected to frequent mechanical activity, such as lungs, skin, or kidneys, also express PIEZO1 (39). The APVAT in rodents is mainly composed of brown adipocytes (BAT). Previous reports describe high expression levels of the PIEZO1 mechanosensor in BAT (20, 40, 41). In addition, preadipocytes cultured under adipogenic conditions maintain their *Piezo1* expression. These results are consistent with previous evidence of the expression of PIEZO1 in preadipocytes, adipocytes, and stem/progenitor cells (18, 19, 28, 29, 33).

### PIEZO1 activation increases intracellular Ca2+ reducing APVAT adipogenic potential

We demonstrated that PIEZO1 activation reduced adipogenesis in APVAT preadipocytes. The chemical agonist Yoda1 was used in this study to determine the role of PIEZO1 since it is highly specific for this mechanosensor (34), avoiding activation of PIEZO2 channels (42, 43). As expected, the expression of *Piezo1* was essential to enhance Ca2+ influx in preadipocytes (**Figure 2**). This observation coincides with those in dental pulp stem cells (DP-MSC) (33), adipocytes (18), and endometrial epithelial cells (35), where Ca^2+^ fluxes shifts are essential to induce mechanoreceptor responses.

The influx of calcium *via* PIEZO1 could alter several functions within the cell, including proliferation, homeostasis, apoptosis, and differentiation (44–46). PIEZO1 activation during early adipogenesis (1 to 4 days post-induction) did not affect cell integrity but impaired differentiation of PVAT preadipocytes. We confirmed these responses using Yoda1 as a pharmacological PIEZO1 agonist and siRNA targeting PIEZO1. The latter reduced *Piezo1* by 70%, a reduction similar to those reported in dental pulp (33) and bone marrow progenitors (29). The Ca2+ flux-mediated anti-adipogenic response to PIEZO1 activation observed in this study is consistent with observations in adipocyte progenitor cells derived from different species and AT sites. Murine and human non-PVAT progenitors had reduced adipogenic potency when exposed to agents that increased intracellular calcium (47, 48) or when the activity of calcium-dependent proteins such as calcineurin, a protein essential for muscle renewal and cardiac hypertrophy development, was increased (49, 50). PIEZO1 activation appears to impair the adipogenic program by reducing the expression of *Pparg*, encoding PPARγ, the master key regulator of adipogenesis which reaches maximum expression during days 3-4 of the adipogenesis process (51). Possibly related to lower PPARγ activity, PIEZO1 activation also reduced the expression of genes relevant for maintaining adipocytes’ phenotype and function, such as *Plin1*, a protein that coats lipid droplets (52); *Adipoq*, a factor highly secreted by adipose tissue that increases PPARγ ligand activity (53); *Agpat2* and *Dgat1*, rate-limiting enzymes for triglyceride synthesis, and *Fabp4* a fatty acid-binding protein (54).

### PIEZO1 activates anti-adipogenic APVAT pathways

In the present study, pharmacological activation of PIEZO1 with Yoda1 elicited expression of *Fgf9* in PVAT preadipocytes. This gene has been identified as a potent inhibitor of adipogenesis and the browning of white adipocytes (55, 56). In addition, *Fgf9* stimulates the proliferation of vascular smooth muscle, epithelial, and colorectal cancer cells (57). The mechanism for this proliferation response is mediated through the activation of the WNT/β catenin pathway (58, 59). Miyazaki et al. demonstrated that pharmacological or mechanical activation of PIEZO1 in dental stem cells upregulates the *Wnt16* member of the Wnt/β catenin family (36). The WNT pathway promotes myogenic differentiation while suppressing adipogenesis (60–64). Remarkably, in the present study, *Wnt16* was upregulated during PIEZO1 activation in APVAT preadipocytes. This may be an alternate mechanism for adipogenesis suppression in APVAT preadipocytes; however, more studies are required to determine if PIEZO1 and *Wnt16* activation can promote myofibrogenesis of these cells during hypertension.

### Mechanical stimulation reduces APVAT adipogenic potential independent of *Piezo1* expression

To our knowledge, this is the first report on the impact of mechanical forces on adipogenesis and lipogenesis in APVAT. Preadipocyte exposure to cyclic mechanical strain during the early stages of adipogenesis reduces adipogenic efficiency, a response that was not affected even when *Piezo1* was silent. This finding suggests that other mechanosensors act parallel with PIEZO1 during mechanosensation in APVAT. The mechanotransduction process can involve different mediators, including mechanosensors and structural proteins. Among mechanosensors, *Piezo2*, *Trpv4*, *Tmem16*, and *Panx1* were identified in APVAT and other PVAT previously; these proteins could complement PIEZO1 mechanosensing activity (20) however, the impact of their activity on adipogenesis remains to be elucidated. Among structural proteins, integrins are a family of transmembrane proteins that can suppress adipogenic genes such as *C/ebpα* and *Ppparg* and promote the expression of anti-adipogenic pathways, including Runx2, β-catenin, and SMAD proteins. Remarkably, integrins can also be activated by Ca2+ influx, a direct response to PIEZO1 activation in APVAT preadipocytes (65). Our results highlight the need for studies evaluating the interaction among PIEZO1, other mechanoreceptors, and structural proteins and their impact on APVAT adipogenesis and function.

### Limitations

Our study provides evidence for the effect of PIEZO1 activation on APVAT preadipocyte adipogenesis. However, given the differences among PVAT sites (e.g., mesenteric PVAT, abdominal aorta PVAT) on adipocyte phenotype and the characteristics of mechanical forces acting on other sites, results from the present study cannot be extrapolated directly to other PVAT tissues. Second, the cyclic mechanical strain frequencies used in the current experiments are lower than those occurring *in vivo*, given their rapid heart rates (66). Thus, future research is needed to establish the direct effect of mechanical force frequency on APVAT. To date the role of PIEZO1 *in vivo* remains elusive, while global deletions lead to embryonic lethality, tissue-specific gain or loss of function demonstrate specific responses depending on the type of cell involved. Therefore, future studies that include targeted deletion of this mechanosensor in preadipocytes and other PVAT cells that take into account sex differences in normotensive and hypertensive animals are required to determine the impact of PIEZO1 on cardiovascular diseases.

## Conclusion

Results from the present study demonstrate that pharmacological activation of the mechanosensory protein PIEZO1 reduces adipogenesis on aortic PVAT preadipocytes. At the same time, mechanical stimulation can activate other routes than PIEZO1 that may suppress adipogenesis. Pharmacological activation of PIEZO1 activation mediated transient calcium influx through the membrane leading to a repression of adipogenic essential genes, which reduces the adipogenic potential. Reduced hyperplasia of adipocytes may diminish the synthesis of vasoactive chemokines, which explains that the progression of hypertension has been associated with loss of anti-contractile response of PVAT (67).

## Data availability statement

The original contributions presented in the study are included in the article/**Supplementary Material**. Further inquiries can be directed to the corresponding author.

## Ethics statement

The animal study was reviewed and approved by Michigan State University Institutional Animal Care and Use Committee.

## Author contributions

CR, GAC, and SW conception and design of the study; CR, JT, EF, and MC performed experiments; CR, GAC, and MC analyzed data; CR, GAC and MC interpreted results of experiments; CR and GAC prepared figures; CR drafted the manuscript; GAC, EF, JT, MC, and SW edited and revised manuscript. All authors contributed to the article and approved the submitted version.

## Funding

This research was funded by NIH NHLBI P01 HL152951.

## Acknowledgments

The authors acknowledge the technical assistance of the Investigative Histopathology lab and all the staff at Contreras and Watts Laboratories at Michigan State University (East Lansing, MI).

## Conflict of interest

The authors declare that the research was conducted in the absence of any commercial or financial relationships that could be construed as a potential conflict of interest.

## Publisher’s note

All claims expressed in this article are solely those of the authors and do not necessarily represent those of their affiliated organizations, or those of the publisher, the editors and the reviewers. Any product that may be evaluated in this article, or claim that may be made by its manufacturer, is not guaranteed or endorsed by the publisher.
