# Supplementary material for: PIEZO1 mechanoreceptor activation reduces adipogenesis in perivascular adipose tissue preadipocytes
Source: Front Endocrinol (Lausanne). 2022 Aug 31;13:995499. doi: 10.3389/fendo.2022.995499 (PMC9471253; doi:10.3389/fendo.2022.995499)
Supplement: Supplementary file 1 [file DataSheet_1.docx]

**
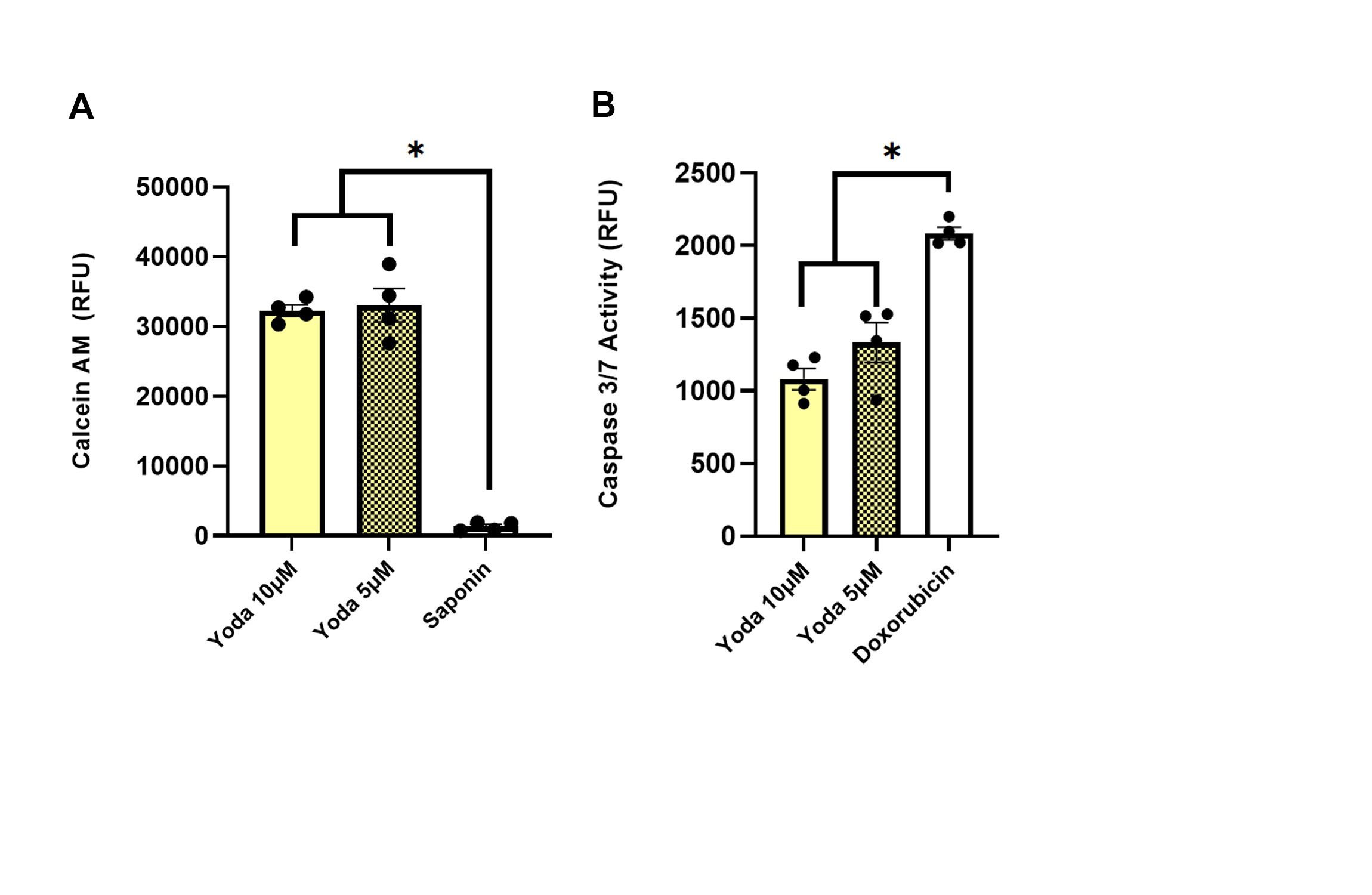
Supplemental figure 1.**

**Supp Figure 1. PIEZO1 chemical activation does not affect preadipocytes viability or apoptosis.** A. Calcein AM signal of induced preadipocytes treated for 4 days with 10µM or 5µM of Yoda1 and 0.1% of Saponin (negative control) B. Caspase 3/7 activity after 4 days of exposure to 10 or 5µM of Yoda1, Doxorubicin HCl was used as positive control. Relative Fluorescence Units (RFU) A. **P*<0.05. representative of n=4.

**Supplemental Table S1.** Primers used for RT-qPCR and siRNA sequences

| Gen | GenBank | Forward Primer | Reverse Primer |
| --- | --- | --- | --- |
| *Actb*^1A^ | NM_031144.3 | AACCGCGAGAAGATGACCCAGATCATGTTT | AGCAGCCGTGGCCATCTCTTGCTCGAACTG |
| *Adipoq* | NM_144744 | CCGTGATGGCAGAGATGG | CTCCTGTCATTCCAGCATCTC |
| *Agpat2* | NM_001106378 | CCTCAAAGTGTGGATTTACC | TTGTAGAAGGAGGAAAAGAG |
| *Col1a1* | NM_053304.1 | TGGATTCCAGTTCGAGTATG | AGTGATAGGTGATGTTCTGG |
| *Dgat1* | NM_053437.2 | TACTCCATCATCTTCCTCAAG | AAGTAATAGAGATCTCGGTAGG |
| *Fabp4* | NM_053365.1 | TCCCTACTTGTGTGGCGTGAA | TCACCCGAGTGGTAGTCACAATG |
| *Fgf10* | NC_051337.1 | ATAACACAGTGGAAATCGG | CTTTTGAGCCATAGAGTTTCC |
| *Fgf2* | NC_051337.1 | AAACTCGGATCCAAAACG | TGTCTAAAGAGAGTCAGCTC |
| *Fgfr1* | NC_051339.1 | CAGACTGGTCTTAGGAAAAG | GCATCAGACTTCAACATCTTC |
| *Fgfr2* | NC_051336.1 | AAGATGTTGAAAGATGACGC | AAGATGTTGAAAGATGACGC |
| *Fgfr3* | NC_051349.1 | CTTTAGTGTGCGTTAACAG | GTTTCTTATCCATACGCTCC |
| *Fn1* | NC_051344.1 | AAGCCAATAGCTGAGAATG | AAGTACAGTCCACCATCATC |
| *Piezo1*^2A^ | NC_051354.1 | GAGGCCTCACAAGGAAAGC | GGGCAGCATCTATGTCATCC |
| *Plin1* | NM_013094 | GTAGAATATCTCCTGCCACCA | TGTGTCGAGAAAGAGTGTTGG |
| *Pparg* | NG_011749 | GGTGTGATCTTAACTGTCGG | TTCAGCTGGTCGATATCACT |
| *Rps29* | X59051 | GCCAGGGTTCTCGCTCTTG | GGCACATGTTCAGCCCGTAT |
| *Smad4* | NC_051353.1 | TACCACCATAACAGCACTAC | GAACACCAATATTCAGGAGC |
| *Wnt16* | NC_051339.1 | GAATCTACACAACAACGAGG | GTTTTGTCTGAGATCTGGATG |
| *Eif3k* | NC_051336.1 | AATTTATCTGCCATGTCGTG | CGATGTTCTTGGGCTTAATG |
| *Rps9* | NC_005100.4 | GATTTCTTGGAGAGAAGACTG | TGAAAGATGGAATGTTCACC |
| *B2m* | NC_051338.1 | ACTGGTCTTTCTACATCCTG | AGATGATTCAGAGCTCCATAG |
| siPiezo1.13.1 | | CAAGAAGUACAAUCAUCUAAACCTC | UGGUUCUUCAUGUUAGUAGAUUUGGAG |
| siPiezo1.13.2 | | CGUCAUCAUCUCUAAGAAUAUGUTG | UGGCAGUAGUAGAGAUUCUUAUACAAC |
| siPiezo1.13.3 | | GCACAGUCAAAGGCUACUAUGACCC | CACGUGUCAGUUUCCGAUGAUACUGGG |

**^1^**Taqman assay (ThermoFisher Cat N Rn00667869_m1)

**^2^**Taqman assay (ThermoFisher Cat N Rn01432593_m1)

**^A^**Data depicted in figure 1B was obtained in the Quan Studio 7 Flex System (Applied Biosystems, MA, USA) in duplicate: each 20 µL PCR reaction contained 1X of PerfeCTa Fast Mix II (Quantabio, Cat N° 95119-012), primers provided at 20X were used at 1X, and 4 ng/µL of sample cDNA.
